# Work and Thermal Fluctuations in Crystal Indentation under Deterministic and Stochastic Thermostats: The Role of System–Bath Coupling

**DOI:** 10.3390/e24091309

**Published:** 2022-09-15

**Authors:** Javier Varillas, Lamberto Rondoni

**Affiliations:** 1 Institute of Thermomechanics, Czech Academy of Sciences, 18200 Prague, Czech Republic; 2 Dipartimento di Scienze Matematiche, Politecnico di Torino, 10125 Turin, Italy; 3 INFN, Sezione di Torino, Via P. Giuria 1, 10125 Turin, Italy

**Keywords:** ensembles, stochastic thermodynamics, work, free energy, mesoscopic systems, absolute irreversibility, non-equilibrium

## Abstract

The Jarzynski equality (JE) was originally derived under the deterministic Hamiltonian formalism, and later, it was demonstrated that stochastic Langevin dynamics also lead to the JE. However, the JE has been verified mainly in small, low-dimensional systems described by Langevin dynamics. Although the two theoretical derivations apparently lead to the same expression, we illustrate that they describe fundamentally different experimental conditions. While the Hamiltonian framework assumes that the thermal bath producing the initial canonical equilibrium switches off for the duration of the work process, the Langevin bath effectively acts on the system. Moreover, the former considers an environment with which the system may interact, whereas the latter does not. In this study, we investigate the effect of the bath on the measurable quantity of the JE through molecular dynamics simulations of crystal nanoindentation employing deterministic and stochastic thermostats. Our analysis shows that the distributions of the kinetic energy and the mechanical work produced during the indentation processes are affected by the interaction between the system and the thermostat baths. As a result, the type of thermostatting has also a clear effect on the left-hand side of the JE, which enables the estimation of the free-energy difference characterizing the process.

## 1. Introduction

The Jarzynski equality (JE) is meant to link the statistics of non-equilibrium works to an equilibrium property: the free-energy difference between two equilibrium states of a given physical system [1]. In this sense, the JE complements the fluctuation–dissipation relationships that obtain non-equilibrium properties from equilibrium experiments. The present literature is vast and substantially includes diverse investigations on the validity of the JE. Most authors report verifications of the theory or provide reasons in support of the validity of the JE; such as those discussing optimal protocols for stochastic systems; see, e.g., Refs. [2,3,4,5]. However, investigations considering a reduced number of work measurements find violations of the JE or express some concern in connection with the Hamiltonian derivation; see, e.g., Refs. [6,7,8,9]. In particular, the study of a variable volume system [9] shows that the Hamiltonian of the JE is not universal. Moreover, Ref. [10] demonstrates that the JE is violated even in small systems complying with the Jarzynski theory due to the emergence of process-dependent irreversibilities at the nano-scale.

In this study, we investigate the effect of deterministic and stochastic thermostats on the measurable (or computable) quantity that appears in the JE. We consider that this assessment is required because there are two fundamentally different derivations of the JE that apparently lead to the same work-fluctuation expression, when in fact they refer to different types of experimental conditions and, hence, to distinct free-energy quantities. To appreciate these differences, which mainly concern the role of thermostatting, we outline in the following the two derivations of the JE.

### 1.1. The Hamiltonian Derivation of the JE

The Hamiltonian derivation of the JE concerns an *N*-particle system (S), with coordinates and momenta expressed by $x=(q_{S},p_{S})$, where the collection of coordinates are $q_{S}=\left(q_{1},\ldots ,q_{N}\right)$ and that of momenta is $p_{S}=\left(p_{1},\ldots ,p_{N}\right)$, that may interact with an environment (E) made of *M* particles, with coordinates and momenta expressed by $y=(q_{E},p_{E})$, where $q_{E}=\left(q_{N+1},\ldots ,q_{N+M}\right)$ and $p_{E}=\left(p_{N+1},\ldots ,p_{N+M}\right)$. We denote by M the phase space of S+E, which is the set of points Γ=(x,y) of all coordinates and momenta of the object constituted by S and E together. The Hamiltonian of S+E is assumed to take the form

(1) $$H\left(\Gamma ;\lambda \right)=H_{S}\left(x;\lambda \right)+H_{E}\left(y\right)+h_{\mathrm{int}}\left(x,y\right)\;,$$

where λ is a time-dependent parameter controlled by an external agent, $H_{S}$ is the energy of S, $H_{E}$ is that of E, and $h_{\mathrm{int}}$ is the interaction energy of S with E. Initially, λ(0)=α, and S+E is in equilibrium with a thermal bath (B) at temperature *T*; hence, the statistics of its phases Γ are given by the canonical ensemble

(2) $$f_{\alpha }\left(\Gamma \right)=\frac{e^{-\beta H(\Gamma ;\alpha )}}{Q_{S+E}\left(\alpha \right)}\;,$$

where

(3) $$Q_{S+E}\left(\lambda \right)=\int e^{-\beta H(\Gamma ;\lambda )}d\Gamma \;,$$

and $\beta ={\left(k_{B}T\right)}^{-1}$ characterizes the thermal bath.

At time t=0, the S+E is disconnected from B, and the external agent acts on λ for a time τ, which varies from its initial value α to its final value λ(τ)=ω. This fully deterministic process is repeated many times, with identical λ(t), but each time starting from a different initial condition $\Gamma_{0}=\left(x_{0},y_{0}\right)$ and different energy $H(\Gamma_{0};\alpha )$ dictated by the initial canonical distribution $f_{\alpha }$. The fact that S+E and B do not exchange energy during the process may be justified by assuming that only a negligible amount of energy can be exchanged in the typically short process time τ. Then, the derivation of the JE—that proceeds through exact analytical calculations—suggests that the work distributions of practically any process taking λ from the given initial value α to the final value ω, during any process time τ>0, can be used to compute the free-energy difference between the canonical state at temperature *T* with Hamiltonian H(Γ;λ(0)=α) and the canonical state at temperature *T* with Hamiltonian H(Γ;λ(τ)=ω). The calculations [1] can be summarized as follows.

Letting $S_{\lambda }^{t}:M\to M$ denote the phase space evolution operator so that Γ∈M evolves into $S_{\lambda }^{t}\Gamma \in M$ at time *t* and letting $x_{t}$ be the coordinates and momenta of S at time *t*, the quantity

(4) $$W_{J}\left[S_{\lambda }^{t}\Gamma ;0\leq t\leq \tau \right]=\int_{0}^{\tau }dt\;\dot{\lambda }\left(t\right)\frac{\partial H_{S}}{\partial \lambda }\left(x_{s};\lambda \left(t\right)\right)$$

is introduced and called *work* [1]. Indeed, when λ represents a position in space and the derivative of $H_{S}$ with respect to λ represents a force, $W_{J}$ corresponds to a mechanical work. Following Ref. [1], one obtains that

(5) $$W_{J}\left[S_{\lambda }^{t}\Gamma ;0\leq t\leq \tau \right]=H\left(S_{\lambda }^{\tau }\Gamma ;\omega \right)-H\left(\Gamma ;\alpha \right)$$

and

(6) $${\langle e^{-\beta W_{J}}\rangle }_{\alpha }=\frac{1}{Q_{S+E}\left(\alpha \right)}\int e^{-\beta W_{J}\left(\Gamma \right)}e^{-\beta H(\Gamma ;\alpha )}\;d\Gamma =\frac{Q_{S+E}\left(\omega \right)}{Q_{S+E}\left(\alpha \right)}=e^{-\beta \left[F_{S+E}\left(\omega \right)-F_{S+E}\left(\alpha \right)\right]}\;,$$

where $\Delta F_{S+E}=\left[F_{S+E}\left(\omega \right)-F_{S+E}\left(\alpha \right)\right]$ is the difference of the free energies of S+E in the canonical equilibrium at temperature *T* with parameters ω and α, respectively. To obtain the free-energy difference essentially related to S, the following quantity is introduced

(7) $$H_{S}^{*}\left(x;\lambda \right)=H_{S}\left(x;\lambda \right)-\frac{1}{\beta }\ln\frac{\int dye^{-\beta \left[H_{E}\left(y\right)+h_{\mathrm{int}}\left(x,y\right)\right]}}{\int dye^{-\beta H_{E}\left(y\right)}}\;,$$

which was proposed by Kirkwood [11] to treat subsystems of macroscopic dense fluids in thermodynamic equilibrium. The quantity $H_{S}^{*}\left(x;\lambda \right)$ is the energy of S plus an average contribution coming from the interaction of S with E. Then, the system whose Hamiltonian is $H_{S}^{*}$, S${}^{*}$ say, can be associated to the canonical ensemble

(8) $$p_{S}^{*}\left(x;\lambda \right)=\frac{e^{-\beta H_{S}^{*}\left(x;\lambda \right)}}{Q_{S}^{*}\left(\lambda \right)}\;;\;Q_{S}^{*}\left(\lambda \right)=\int dx\;e^{-\beta H_{S}^{*}\left(x;\lambda \right)}$$

and to the free energy

(9) $$F_{S}^{*}\left(\lambda \right)=-\beta^{-1}\ln Q_{S}^{*}\left(\lambda \right)\;,$$

i.e., the free energy of a hypothetical system with Hamiltonian $H_{S}^{*}$. This quantity is then linked to the *solvated* free energy of “*S in E*” [12].

It is found that

(10) $$\Delta F_{\alpha \to \omega }^{*}\equiv \left[F_{S}^{*}\left(\omega \right)-F_{S}^{*}\left(\alpha \right)\right]=\left[F_{S+E}\left(\omega \right)-F_{S+E}\left(\alpha \right)\right]\;,$$

which readily leads to the JE

(11) $${\langle e^{-\beta W_{J}}\rangle }_{\alpha }=e^{-\beta \Delta F_{\alpha \to \omega }^{*}}\;,$$

where ${\langle \cdot \rangle }_{\alpha }$ is the canonical average with respect to the initial ensemble $f_{\alpha }$ of S+E. As noted in Ref. [12], transformations of the free energy of a system strongly interacting with another one are not particularly interesting; hence, some kind of solvated free energy is preferred.

There are two aspects of this theory that seem to remain unscrutinized in the present literature. First, the energy exchange between S+E and B may be small, but so is the free-energy variation computed by means of the JE. Moreover, E can be absent in the Hamiltonian derivation or, equivalently, $h_{\mathrm{int}}$ may vanish. This is the case of the Langevin derivation, outlined in the following section. However, there is a second major difference between the two derivations: B acts on S during the Langevin process, whereas in the Hamiltonian derivation B is not considered.

### 1.2. The Langevin Derivation of the JE

The JE can also be derived under a stochastic framework [13], particularly using the Langevin equation [2,4]

(12) $$dX_{t}=F\left(X_{t},\lambda \left(t\right)\right)dt+\sqrt{\frac{2}{\beta }}dW_{t}\;,\;F\left(x,\lambda (t)\right)=-\partial_{x}U\left(x,\lambda (t)\right)\;,$$

where $X_{t}$ is the vector of position coordinates of S, *U* is a time-dependent potential with t∈[0,τ], and $W_{t}$ is a Wiener process. Then,

(13) $$w_{t}=\int_{0}^{\tau }\partial_{\lambda }U\left(X_{t},\lambda \left(t\right)\right)\dot{\lambda }\left(t\right)\;dt$$

is a quantity related to the variations of the system’s energy produced by changes in λ. In the particular case in which λ represents a position in space, $w_{t}$ becomes a mechanical work. Additionally, when λ defines the position of the external agent exerting a force *F* on the system, $w_{t}$ describes the work carried out by the force (−F) acting on the agent. Because there is no environment, the relevant free energy is that of S alone, which can be called *intrinsic* free energy. At a given λ, this is defined by

(14) $$G\left(\lambda \right)=-\frac{1}{\beta }\log\;Z\left(\lambda \right)\;,$$

where

(15) $$Z\left(\lambda \right)=\int_{\Omega }\;e^{-\beta U(x,\lambda )}\;dx\;,$$

and Ω is the set of coordinates *x*. The quantity G(λ) is *intrinsic* in the sense that it solely refers to the properties of the system S. Thus, this framework is analogous to that adopted in the deterministic Hamiltonian derivation assuming weak coupling ($h_{\mathrm{int}}\approx 0$). However, unlike the Hamiltonian derivation, the stochastic Langevin formulation allows S to interact with B assuming that the kinetic energy plays no explicit role in the work statistics.

To obtain the JE, one can start from the joint random variable $\left(X_{t},w_{t}\right)$ and its probability density $p_{t}\left(x,a\right)$, where a∈R. Then, the quantity of interest

(16) $${\tilde{p}}_{t}\left(x\right)=\int_{-\infty }^{\infty }\;da\;e^{-a}p_{t}\left(x,a\right)$$

with initial condition

(17) $${\tilde{p}}_{0}\left(x\right)=\frac{e^{-\beta U(x,\lambda (0))}}{Z(\lambda (0))}$$

obeys

(18) $${\tilde{p}}_{t}\left(x\right)=\frac{1}{Z(\lambda (0))}\;\exp\left[-\beta U(x,\lambda (t))\right]\;.$$

Because the average of the exponential of $-w_{t}$ with respect to the initial distribution is expressed through

(19) $$E_{0}\left(e^{-w_{\tau }}\right)=\int_{\Omega }\;dx\int_{-\infty }^{\infty }\;da\;e^{-a}p_{\tau }\left(x,a\right)=\int_{\Omega }\;dx\;{\tilde{p}}_{\tau }\left(x\right)\;,$$

by taking λ(0)=α and λ(τ)=ω, the JE is readily obtained

(20) $$E_{0}\left(e^{-w_{\tau }}\right)=\frac{Z(\omega )}{Z(\alpha )}=\exp\left\{-\beta \left[G(\omega )-G(\alpha )\right]\right\}.$$

Analogous to the Hamiltonian formulation, the JE is here derived irrespective of the form of λ(t), as long as λ(0)=α and λ(τ)=ω, for any process time τ. Note that Equation (20) is the JE for the intrinsic—not the solvated—free-energy difference of S. Thus, when E is absent (in the Hamiltonian formulation), both derivations refer to the same intrinsic quantity.

### 1.3. Comparison between the Two Derivations

The above summaries show that the Hamiltonian and Langevin theoretical derivations refer to two different classes of experiments. The former refers to systems that do not interact with a heat bath for the duration of the work process, whereas the latter to systems that do interact with a thermal bath. The described experiments differ also in assuming the presence or the absence of a third object, called environment (here, denoted as E). The conditions in which E acts on S can be related to protein stretching experiments [14], where S can be represented by the protein, E by the water in which the protein is immersed, and B can be the air of the laboratory in which the water pool is situated. The Langevin setting may also involve an environment E. However, the interaction of E with S is reduced due to the viscosity damping applied to S and not to an effective interaction energy ($h_{\mathrm{int}}$ from Equation (1)). This is the case of the harmonic oscillators reviewed—along with other experimental settings—in Ref. [15].

In both derivations, the result is process independent. The protocol independence is less obvious under the Hamiltonian framework, where rapid transformations may not allow an efficient exchange of energy between S and B, and the number of particles as well as space and time scales strongly influence the system’s response to external perturbations.

We consider that the role of system–bath coupling in the work statistics—which is the measurable quantity in the JE—requires further evaluation. Assuming a condition in which E is absent, both Hamiltonian and Langevin derivations of the JE lead to the intrinsic free-energy variation and follow the same expression. The fact that nonequilibrium processes typically last only a short time supports that the exchanged energy may be negligible. However, this is a delicate assumption in at least two regards: (1) the values of the free-energy difference are usually small as well, and (2) the process independence convoked by both approaches allows the process time to be long.

To investigate such questions, we consider the molecular dynamics model of crystal nanoindentation described in Ref. [10] using Nosé–Hoover and Langevin thermostats. Our analysis shows that the the coupling between S and B has a clear effect on the resulting distributions of the kinetic energy and the mechanical work. Consequently, the left-hand side of the JE is affected. Interestingly, the work statistics obtained in indentations protocols that produce fully reversible elastic deformations in the crystal lead to different distributions as a function of the thermostat.

## 2. Computational Methods

### 2.1. Molecular Dynamics Setup: Crystal Nanoindentation

We carry out an extensive number of molecular dynamics (MD) simulations to investigate the properties of a minute (001)-oriented Ta crystal indented by a spherical nanoscopic tip. To run the simulations, we employ the open-source LAMMPS code [16]. The interatomic force field is modeled by means of the embedded-atom method potential developed by Ravelo et al. [17].

The Ta crystal has a cuboid shape of size 10.8×10.8×6nm${}^{3}$ and contains 40,293 particles. In the MD domain, the crystal’s particles are sorted into two groups: the particles contained in the two lowermost atomic planes of the crystal create an effective floor that prevents the downward displacement of the crystal during indentation as their motion is restricted. The remaining particles constitute the hereafter called *particle system*, which includes a total number of *N* = 39,204 particles whose positions, q ≡ $\left\{q_{1};q_{2};\ldots ;q_{N}\right\}$, and velocities, v ≡ $\left\{v_{1};v_{2};\ldots ;v_{N}\right\}$, are, respectively, denoted by $q_{i}$ = $\left(x_{i},y_{i},z_{i}\right)$ and $v_{i}$ = $\left(v_{ix},v_{iy},v_{iz}\right)$ for *i* = 1,2,…,N. The system’s particles are free to move and interact with each other according to the prescribed ensemble properties and the interatomic potential. Periodic boundaries are applied to the lateral sides of the crystal.

The indenter is modeled by a spherical-shaped repulsive potential,

(21) $$\Phi \left(q,q_{c}\right)=\sum_{i=1}^{N}\varphi \left(q,q_{c}\right)\;;\;\varphi \left(q_{i},q_{c}\right)=\left\{\begin{array}{ccc} -k\;\delta_{i}^{3}/3 & & \;\;\delta_{i}\leq 0\;\;\\ 0 & & \;\;\delta_{i}>0\;, \end{array}\right.$$

of radius *R* = 3 nm and center C with coordinates $q_{c}=\left(x_{c},y_{c},z_{c}\right)$, where *k* is the indenter stiffness and $\delta_{i}={\left[{\left(x_{i}-x_{c}\right)}^{2}+{\left(y_{i}-y_{c}\right)}^{2}+{\left(z_{i}-z_{c}\right)}^{2}\right]}^{0.5}-R$. Note that the indenter acts on particle *i* when $\delta_{i}\leq 0$. Then, the repulsive force exerted on particle *i*, $F_{i}=-\left(\partial \varphi /\partial q_{i}\right)$, takes

(22) $$\left(F_{ix},F_{iy},F_{iz}\right)=\left\{\begin{array}{ccc} k\delta_{i}^{2}\left(x_{i}-x_{c},y_{i}-y_{c},z_{i}-z_{c}\right)/\left(\delta_{i}+R\right) & & \delta_{i}\leq 0\;\;\\ 0 & & \delta_{i}>0\;. \end{array}\right.$$

Here, *k* is set to 100 eV/Å${}^{3}$. Notice in Equation (22) that the direction of the repulsive force from the indenter is dictated by the $\left(q_{i}-q_{c}\right)$ vector.

**Figure 1:**
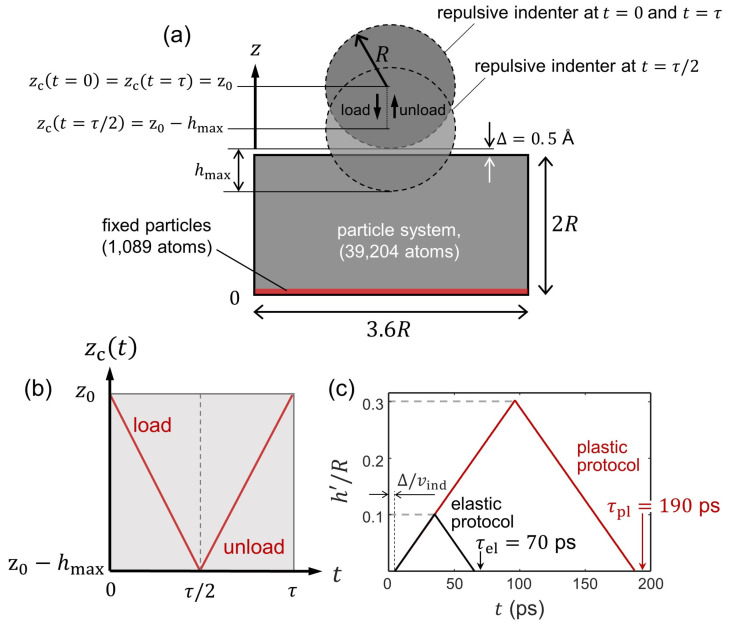
Simulation setup. (**a**) Schematic representation of the indentation process. The computational domain contains the particle system of size 3.6R×3.6R×2R and the repulsive spherical indenter of radius *R* modeled by Equation (21). The vertical coordinate of the indenter center, $z_{c}$, is plotted in (**b**) as a function of the process time τ. (**c**) The evolution of the normalized effective penetration, ${\left(h^{\prime }/R\right)}_{\max}$ where $h^{\prime }=h-\Delta$, as a function of *t* for both elastic and plastic cases with $v_{\mathrm{ind}}$ = 10 m/s.

Figure 1a depicts the computational domain in our MD simulations, which contains the Ta crystal and the repulsive indenter. This computational approach has been largely employed in MD studies of indentation in metallic bodies (cf. Ref. [18] and references therein).

We create an initial state in which the particles occupy the lattice positions of the Ta crystal and the velocities are taken from a normal distribution with 0 mean and a standard deviation scaled to produce a temperature T=300 K. To generate an equilibrium canonical distribution at *T*, we run a preliminary 20 ps thermalization during which the particles follow *NVT* conditions with the Nosé–Hoover (NH) thermostat [19] controlling the system’s temperature at T=300 K. Thus, the positions and velocities of the particles at t=0 (i.e., prior to indentation) are sampled from the canonical distribution produced during the *NVT* thermalization run.

The indentation run consists of a closed loading/unloading loop where the indenter moves vertically with constant velocity, $v_{\mathrm{ind}}$ = 10 m/s. The motion of the vertical coordinate of the indenter center, $z_{c}$, is described through

(23) $$z_{c}\left(t\right)=z_{0}-h\left(t\right)\;,\;h\left(t\right)=\left\{\begin{array}{cc} v_{\mathrm{ind}}\;t & \;\;\;t\in [0,\tau /2)\;\;\\ \;\;\;\;v_{\mathrm{ind}}\;\left(\tau -t\right) & \;\;\;\;t\in [\tau /2,\tau ]\;, \end{array}\right.$$

where h(t) maps the penetration of the indenter during τ. Note that the maximum penetration, $h_{\max}$, is attained at t=τ/2, where $z_{c}\left(t=\tau /2\right)$ = $z_{0}-h_{\max}$ and τ = $2h_{\max}/v_{\mathrm{ind}}$; see Figure 1b. We conveniently define $z_{0}$ such that the indenter tip and the top surface of the crystal are separated by a small vertical distance Δ=0.5 Å, so that the constituting particles are guaranteed to lie outside of the radius of action of Φ at *t* = 0 and *t* = τ; see Figure 1a. This also allows the particles to arrange initially into an unperturbed Ta bcc crystalline configuration during the thermalization run. The applied indentation load, *P*, is then defined as the sum of the vertical, repulsive force contribution, $P=-\sum_{i=1}^{N}F_{iz}$, coming from the particles that satisfy $\delta_{i}\leq 0$; see Equation (22). The computational timestep, d*t*, is set to 2 fs in all of the MD simulations.

In our indentation setting, the particles of the crystal constitute the system of interest S, the indenter—whose time dependent potential energy appears in the Hamiltonian of S—represents the external agent, and no environment is included, so $h_{\mathrm{int}}$ = 0. The Hamiltonian of S is then given by

(24) $$H\left(q,p,q_{c}\right)=K\left(p\right)+V\left(q\right)+\Phi \left(q,q_{c}\right)\;,$$

where q is the vector of position coordinates; p=mv is the vector of momenta of all particles, with *m* being the mass of a Ta atom and v being the set of their velocities; K(p) is the kinetic energy of the system (Section 2.2.2); and V(q) is the potential energy of the interatomic interactions. Note that, here, the time-dependent parameter of the Jarzynski theory is $\lambda \left(t\right)=q_{c}\left(t\right)$, i.e., the moving center of the indenter potential Equation (21).

To evaluate the effect of the coupling between the system and the bath on the mechanical and kinetic response of the system to the indentation processes, we adopt the following computational approaches.

(1) We perform MD indentation simulations using the (deterministic) NH thermostat with 3 NH chains [19] to implement a condition of constant number of particles *N*, volume *V*, and temperature (that represents the thermal bath at *T* = 300 K). To tune the coupling of the particles with the NH bath, we vary the NH thermostat parameter $\omega_{p}$ that accounts for the frequency at which the particles are thermostatted; see the discussion given in Supplementary Section S1. With $\omega_{p}$ = 100 d*t*, the energy exchange between the system and the NH bath is sensibly strong despite the short time of our indentation processes; see Supplementary Figure S1.
(2) For $\omega_{p}$ = 100,000 d*t*, the sluggishness of the heat flow between the system’s particles and the NH bath describes similar conditions to those considered in the Jarzynski theory, which neglects the system–bath coupling.
(3) By removing the thermostat—i.e., the $\omega_{p}$ = *∞* limit—we obtain an adiabatic evolution of the system with unthermostatted particles. These conditions emulate those considered in the Hamiltonian derivation of the JE.
(4) Lastly, we use a stochastic Langevin thermostat at *T* = 300 K, which acts on the system via a random force [20]. We impose a damping coefficient of $\gamma_{L}$ = 1 ps${}^{-1}$ that allows an efficient energy exchange between the system and the Langevin bath. For further details, see Supplementary Section S2. This approach reproduces the scheme adopted in the Langevin derivation of the JE.

### 2.2. Computation of Thermodynamic and Mechanical Properties

Classical MD provides a window into the microscopic dynamical behavior of the constituent particles of the system. Thus, MD simulations—unlike experiments—give access to all the necessary dynamical ingredients of particle systems, which enables the computation of equilibrium macroscopic properties by sampling from a statistical mechanical ensemble.

In this investigation, special attention is given to two specific quantities measured during the indentation runs (during which Φ effectively acts on the system): (i) the mechanical work exerted to the indenter by the particles and (ii) the total kinetic energy of the system’s particles. In the following, we explain the post-simulation and on-the-fly algorithms that we employ to access to these quantities.

#### 2.2.1. The Mechanical Work

The elementary mechanical work carried out on the indenter by the system,

(25) $$dW_{S}=\sum_{i=1}^{N}\left(-F_{iz}\right)dz_{c}=-\sum_{i=1}^{N}\frac{k\;\delta_{i}^{2}\;\eta \left(\delta_{i}\right)}{\delta_{i}+R}\;\left(z_{i}-z_{c}\right)\;dz_{c}\;,$$

involves the infinitesimal displacements of the indenter, $dq_{c}=\left(0,0,dz_{c}\right)$ and the opposite vertical forces, $-F_{iz}$, where η is the step function, so $\eta (\delta_{i})=1$ for $\delta_{i}\leq 0$ and $\eta (\delta_{i})$ = 0 for $\delta_{i}>0$. (As discussed in Ref. [10], the elementary mechanical work carried out by the indenter on the system, d$W_{I}$ = $\sum_{i=1}^{N}F_{i}\cdot dq_{i}$, differs not only in the sign from Equation (25) but also substantially. Thus, an external operator cannot obtain the work carried out on the system $W_{I}$ from external measurements of d$W_{S}$, particularly in small systems made of classical particles). Note that the negative sign in Equation (25) comes from the force that particle *i* exerts on the indenter as derived from the action–reaction principle. Because $-F_{iz}\geq 0$, the term d$z_{c}$ dictates the sign of d$W_{S}$. Additionally, note that d$z_{c}$ = $-v_{\mathrm{ind}}$d*t* during the loading stage and d$z_{c}$ = $+v_{\mathrm{ind}}$d*t* during unloading; see Equation (23).

We use an in-house post-simulation code to obtain d$W_{S}\left(q,q_{c};t\right)$ by means of Equation (25). This equation is evaluated under evenly spaced infinitesimal time intervals of δt = 0.05 ps that corresponds to a net (infinitesimal) variation in the vertical indenter motion of 5 pm (=0.05 Å). Finally, the total mechanical work $W_{S}$ carried out during a time interval from t = 0 to t = τ is calculated as the sum of the computed elementary works from Equation (25),

(26) $$W_{S}\left(\tau \right)=dW_{S,1}+dW_{S,2}+\ldots +dW_{S,l}\;,$$

where *l* = τ/δt. In our MD indentation setup, $W_{S}\left(\tau \right)$ = $-W_{J}\left(\tau \right)$ [10].

#### 2.2.2. The Total Kinetic Energy

We also obtain on-the-fly values of the total kinetic energy of the particle system,

(27) $$K_{\mathrm{tot}}=\sum_{i=1}^{N}K_{i}=\frac{1}{2}\sum_{i=1}^{N}m\;v_{i}^{2}=\frac{1}{2}\sum_{i=1}^{N}m\;\left(v_{ix}^{2}+v_{iy}^{2}+v_{iz}^{2}\right)\;,$$

where $K_{i}$ is the instantaneous, *translational* kinetic energy of particle *i*.

To investigate the effect of the system–bath coupling on the system’s thermodynamic properties during indentation, we assess the kinetic fluctuations of the system in terms of the statistical behavior of the quantity $K_{\mathrm{tot}}$. In our indentation simulations, $K_{\mathrm{tot}}$ is evaluated every 0.05 ps (=25 d*t*). Since we perform swift nonequilibrium processes and our MD simulations refer to a relatively small number of particles ($N=O(10^{4})$), the consequences of the thermodynamic limit can be observed. In particular, note that $K_{\mathrm{tot}}$ might not coincide with $3Nk_{B}T/2$, where *T* is the bath’s temperature. When $K_{\mathrm{tot}}$ is averaged over the ensemble of initial conditions, we refer to this quantity as $\langle K_{\mathrm{tot}}\rangle$.

## 3. Results and Discussion

In this study, we present a statistical analysis of the work and the kinetic energy obtained during MD indentations performed in absence as well as in presence of thermal baths modeled by NH and Langevin thermostats. Attention is also given to the effect of the thermostat coupling on the left-hand side of the JE (Equation (11)), which is the measurable quantity in the Jarzynski theory. To this end, we consider two distinct indentation protocols that lead to different perturbations of the system. The load/unload indentation protocols are characterized in terms of maximum indenter penetration, $h_{\max}$, attained at *t* = τ/2. The dynamics of the particle system are defined according to the four distinct sampling methods described in Section 2.1. This gives us eight different indentation simulations. Each protocol is repeated over a large number of realizations (*n* = 1000). The individual realizations of the process corresponds to a different initial condition (at *t* = 0) drawn from the canonical distribution (2), produced during the *NVT* thermalization run during which the indenter potential Φ from Equation (24) is effectively 0; see Section 2.1.

### 3.1. Indentations with Elastic Deformations

**Figure 2:**
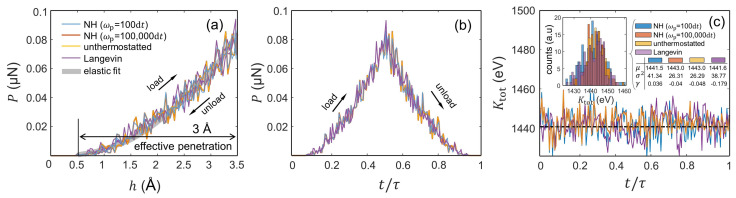
Single realizations of the elastic protocol ($h_{\max}$ = 3.5 Å) with four different system–bath thermostat couplings. The Hamiltonian of the particle system prior to indentation, $H(q,p,q_{c}\left(0\right))$, is identical in each realization. (**a**,**b**): P−h and P−t/τ curves, respectively. In (**a**), the overlap of the load and unload paths manifests reversibility in the elastic protocols. The gray line in (**a**) represents the elastic fit anticipated by the Hertzian contact theory, where *P*∼${\left(h^{\prime }\right)}^{3/2}$. (**c**) $K_{\mathrm{tot}}$ time evolution. The dashed horizontal line in (**c**) marks the kinetic temperature, $K_{\mathrm{tot}}^{\prime }=(3Nk_{B}T$)/2, expected with *T* = 300 K and *N* = 39,204 atoms. The inset to (**c**) shows the corresponding $K_{\mathrm{tot}}$ distributions characterized by the first three central moments (see the table), where μ is the mean (in eV), $\sigma^{2}$ is the variance (in eV${}^{2}$), and γ is the skewness of the $K_{\mathrm{tot}}$ data.

The load/unload indentations with fixed maximum penetration $h_{\max}$ = 0.1R+Δ = 3.5Å characterize the herein called *elastic protocol*; see Figure 1c. Our MD indentations with $h_{\max}$ = 3.5Å result in perturbations of the crystal that lead to elastic contacts between the indenter and the crystal’s surface (Supplementary Figure S3), where the resulting P−h curves shown in Figure 2a follow a good agreement with the continuum elastic behaviour predicted by the Hertzian contact theory [21]. In this context, Figure 2a,b show that the unload stage approximately traces back the mechanical load path followed during loading, which suggests that the process is fully reversible. Additionally, notice that the load–unload curves are unaffected by the presence or absence of a thermostat. Interestingly, although the adiabatic and weakly thermostatted indentation are sampled from fundamentally different statistical ensembles, the corresponding *P* and $K_{\mathrm{tot}}$ evolutions are practically identical; see the inset in Figure 2c. To confirm reversibility, Figure 2c further shows that the total kinetic energy of the particle system fluctuates around a constant $K_{\mathrm{tot}}$ value irrespective of the thermostat coupling, even under unthermostatting conditions where there is strictly no coupling.

**Figure 3:**
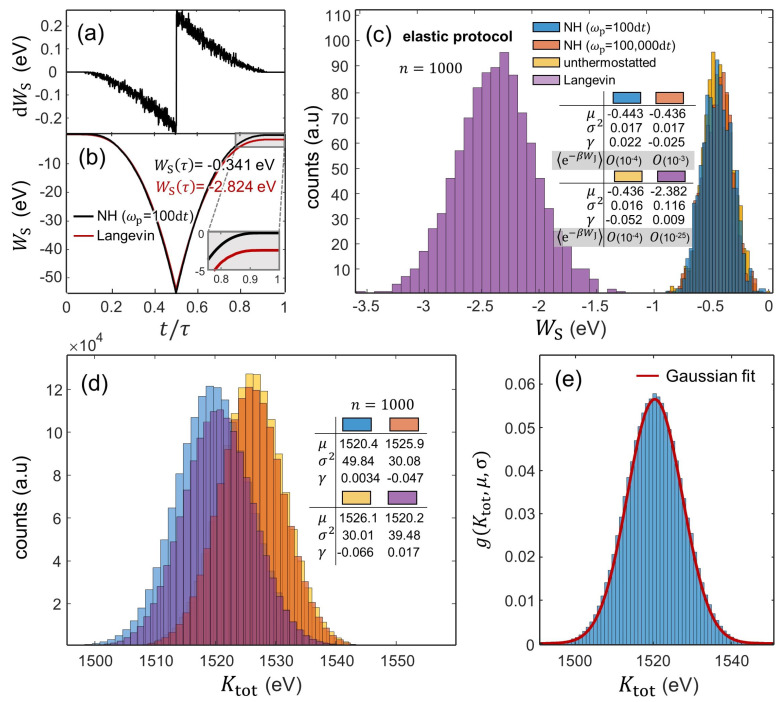
Work and kinetic energy fluctuations as a function of the thermostat coupling during the elastic protocol. (**a**,**b**): Evolution of d$W_{S}$ and $W_{S}$ in terms of t/τ. The plots feature single realizations using NH-thermostatted ($\omega_{p}$ = 100 d*t*) particles in (**a**) and NH- and Langevin-thermostatted particles in (**b**). (**c**) $W_{S}$ histograms from 1000 realizations. $W_{S}$ stands for $W_{S}(t$ = τ) obtained by means of Equation (26). (**d**) $K_{\mathrm{tot}}$ histograms from 1000 realizations. The Gaussian approximation of the $K_{\mathrm{tot}}$ PDF—which describes near equilibrium processes—from the indentations with NH-thermostatted ($\omega_{p}$ = 100 d*t*) particles is given in (**e**).

Reversibility in the indenter-induced elastic perturbation is also evident in the work evolutions shown in Figure 3a,b. Along the time interval [0,τ], $W_{S}\left(t\right)$ gradually decreases from 0 to its minimum value at *t* = τ/2, and then, $W_{S}\left(t\right)$ increases during unloading, approximately matching the loading $W_{S}-h$ path; see Figure 3b. Note that the discontinuity in Figure 3a stems from the fact that the indenter’s motion is inverted at *t* = τ/2 whereas the forces exerted by the indenter at *t* = ${\left(\tau /2\right)}^{-}$ and at *t* = ${\left(\tau /2\right)}^{+}$ remain identical.

Figure 3c shows the $W_{S}$ histograms from *n* = 1000 realizations of the indentations with $h_{\max}$ = 3.5Å, which reveals fundamental details regarding the work fluctuations as a function of the thermostat coupling. We find that all $W_{S}$ distributions nearly adhere to normal distributions with values of skewness, γ, close to 0. In addition, the indentations with varying NH thermostat parameter $\omega_{p}$ (namely with $\omega_{p}$ = 100 d*t* and $\omega_{p}$ = 100,000 d*t*) produce similar $W_{S}$ distributions with μ ≈ −0.44 eV and $\sigma^{2}$ ≈ 0.016 eV${}^{2}$. However, the $W_{S}$ data from the indentations with Langevin-thermostatted particles substantially differ from the other indentations, where the resulting $W_{S}$ distribution becomes considerably wider (with $\sigma^{2}$ = 0.116 eV${}^{2}$) and shifts toward more negative values (with an average value of μ ≈ −2.4 eV). We attribute this to the damping induced by the Langevin thermostat, which hinders the (elastic) recovery to the initial state. This is evidenced in the $W_{S}-t/\tau$ evolutions drawn in Figure 3b, which capture the gradual divergence of the $W_{S}$ values as *t* → τ with Langevin-thermostatted particles as compared with that with NH-thermostatted particles (with $\omega_{p}$ = 100 d*t*). (Note that the difference between these $W_{S}(t$ = τ) values exists because it exceeds the statistical deviation of the $W_{S}$ data from the indentations with NH-thermostatted particles. Additionally, note that a return to the initial state would require quasi-static transformations during which an efficient exchange of energy between the S and E occurs and $W_{S}\left(\tau \right)=0$; see Ref. [10]. Clearly, these conditions are easier to obtain with Langevin baths as they tend to exchange energy with the system more efficiently than the NH thermostats that we employ).

In light of these results, we also find that the values of the measurable quantity in the JE, $\langle e^{-\beta W_{J}}\rangle$, are affected by the thermostatting coupling; cf. the table in Figure 3c.

The plots of Figure 3d show the $K_{\mathrm{tot}}$ histograms as a function of the thermostat coupling. We also find that these indentations produce normal $K_{\mathrm{tot}}$ distributions with values of the skewness, γ, close to 0. The probability density function (PDF) describing such normally distributed $K_{\mathrm{tot}}$ datasets can then be approximated by the general form of the Gaussian function, $g\left(K_{\mathrm{tot}},\mu ,\sigma \right)={\left(\sigma \sqrt{2\pi }\right)}^{-1}\;\exp\left[-\left(K_{\mathrm{tot}}-\mu^{2}\right)/2\sigma^{2}\right]$; see the normalized histogram in Figure 3e. Contrarily to the $W_{S}$ distributions, the $K_{\mathrm{tot}}$ histograms render similar values of the average (μ ≈ 1520 eV) and the variance ($\sigma^{2}$ ≈ 30–40 eV${}^{2}$) regardless of the imposed thermostat coupling. Nonetheless, the indentation processes performed with a weak NH thermostat ($\omega_{p}$ = 100,000 d*t*) and with unthermostatted particles statistically produce distributions marginally shifted toward larger $K_{\mathrm{tot}}$ values as compared with those with thermostatted particles; cf. Figure 3d.

### 3.2. Indentations with Plastic Deformations

With deeper indenter penetrations than those produced by the elastic protocol, the mechanical response changes drastically. In contrast to the reversible elastic deformations discussed in the previous section, indentations with $h_{\max}>4.5\;$Å induce in the crystal non-reversible plastic deformations that persist over time. In general terms, crystal plasticity allows metals to sustain deformations beyond the elastic limit through the formation of non-reversible crystalline distortions. In the case of indented metallic crystals, plasticity manifests through the generation of crystalline defects under the indenter tip; see Supplementary Figure S4 and also Ref. [18]).

The indentations concerning a load/unload process with a maximum value of the indenter penetration of $h_{\max}$ = 0.3R+Δ = 9.5Å characterize the herein called *plastic protocol*; see Figure 1c. In these indentations, the transition to a plastic stage is attained at penetrations of $h\approx h_{\max}/2$. Hence, the imposed $h_{\max}$ is well within the penetration range in which plastic features can be readily observed in the P−h curves.

**Figure 4:**
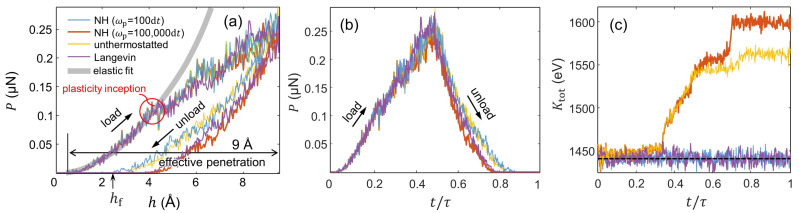
Single realizations of the plastic protocol ($h_{\max}$ = 9.5 Å) with the four different thermostat couplings. The Hamiltonian of the particle system prior to indentation, $H(q,p,q_{c}\left(0\right))$, is identical in each realization. (**a**,**b**): P−h and P−t/τ curves, respectively. The process exhibits the emergence of irreversibility due to the indenter-induced plastic deformations in the crystal evidenced by the utterly mismatched load and unload paths. The gray line in panel (**a**) represents the elastic fit given by the Hertzian contact theory, where *P*∼${\left(h^{\prime }\right)}^{3/2}$. Note in (**a**) that departure from the elastic behavior (highlighted with a red circle) manifests the inception of plasticity. (**c**) $K_{\mathrm{tot}}-t/\tau$ evolution during the indentations with $h_{\max}$ = 9.5 Å. The dashed horizontal line in (**c**) marks macroscopic kinetic temperature, $K_{\mathrm{tot}}^{\prime }=(3Nk_{B}T$)/2, expected with *T* = 300 K and *N* = 39,204 atoms.

The *P*-*h* curves from Figure 4a are characterized by an early elastic response followed by a marked load drop at the inception of plasticity. (Similar drops are observed in DNA pulling experiments [14]). With increasing penetration, further load drops result from the activation of additional plastic processes in the crystal. The P−h evolution then diverges from the elastic fit, thus manifesting the emergence of *irreversibility* in the system. Note that, unlike in the elastic protocol, the onset of plasticity leads to a marked increase in the instantaneous kinetic energy in the indentations with weakly NH-thermostatted ($\omega_{p}$ = 100,000 d*t*) and unthermostatted particles; see Figure 4c. During unloading, the force vanishes at effective penetration values greater than 0 (or $h_{f}>\Delta$ in Figure 4a). In this regard, steep unloading curves are a fundamental manifestation of the formation of a plastic imprint during the indentation process, which remains in the particle system upon removal of the indenter tip from the crystal’s surface. (For further details of such indentation responses in metals using nanometer-sized indenter tips, see Refs. [18,22]).

**Figure 5:**
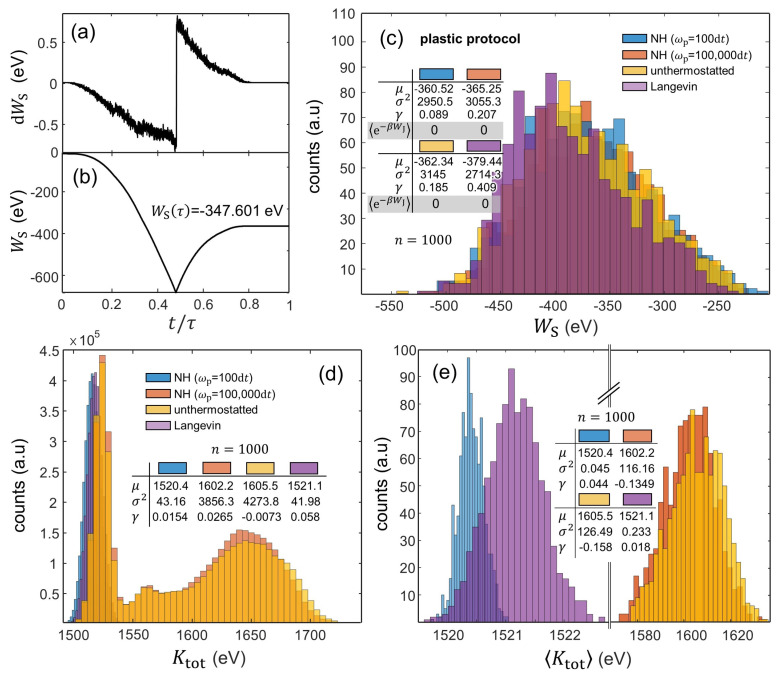
Work and kinetic energy fluctuations as a function of the thermostat coupling during the plastic protocol. (**a**,**b**): Evolution of d$W_{S}$ and $W_{S}$ in terms of t/τ. The plots concern a single realization using NH-thermostatted particles. (**c**): $W_{S}$ histograms from 1000 realizations. $W_{S}$ stands for $W_{S}\left(t=\tau \right)$ obtained by means of Equation (26). (**d**,**e**): $K_{\mathrm{tot}}$ and $\langle K_{\mathrm{tot}}\rangle$ histograms from 1000 realizations. The quantity $\langle K_{\mathrm{tot}}\rangle$ corresponds to the averaged value of $K_{\mathrm{tot}}$ over τ obtained for each individual realization.

Irreversibility also becomes manifest in the $W_{S}$ time evolution obtained during the plastic protocol, where the load stage produces a greater absolute value of $W_{S}$ than during unloading (notice that $W_{S}$ takes a negative value over the [0,τ/2) time interval and a positive value over [τ/2,τ]); see Figure 5a,b. As a result, indentations with $h_{\max}$ = 9.5Å systematically lead to negative $W_{S}$; see Figure 5c. Interestingly, our analysis indicates that the $W_{S}$ histograms exhibit relatively similar moments irrespective of the thermostat coupling, with an expected $W_{S}$ value of ≈−370 eV and a variance of $\sigma^{2}$ ≈ 3000 eV${}^{2}$. Somewhat unexpectedly, the $W_{S}$ distribution from the indentations with Langevin-thermostatted particles slightly differs from the deterministic approaches, as it only exhibits some left-hand skewness (γ ≈ 0.4). Our results from the plastic protocol indicate that the the left-hand side of the JE is uncomputable as $\langle e^{-\beta W_{J}}\rangle \to 0$; see the table in Figure 5c).

The resulting thermal fluctuations obtained in the plastic protocol are assessed through the $K_{\mathrm{tot}}$ and $\langle K_{\mathrm{tot}}\rangle$ distributions shown in Figure 5d,e, respectively. Upon the occurrence of plastic deformations in the crystal, the $\langle K_{\mathrm{tot}}\rangle$ distributions from the indentations with strong system–bath coupling substantially differ in terms of the employed thermostat. Moreover, the indentations with a strong coupling show that the simulations using the Langevin thermostat lead not only to a wider normally distributed $\langle K_{\mathrm{tot}}\rangle$ histogram than that obtained with the NH thermostat (with $\sigma^{2}$ = 0.223 eV${}^{2}$ and 0.045 eV${}^{2}$, respectively) but also to statistically larger values of $\langle K_{\mathrm{tot}}\rangle$; see Figure 5d. This essentially highlights the differences in performance of stochastic vs. deterministic thermostats. On the other hand, the indentations with the system weakly coupled to the thermal bath produce $K_{\mathrm{tot}}$ bimodal distributions as a result of the thermal fluctuations obtained before and after the onset of plasticity in the crystal. In addition, when thermostatting is effectively inoperative in the plastic protocol, the thermal fluctuations during indentation become markedly wild, and thus, the $\langle K_{\mathrm{tot}}\rangle$ distributions shift toward larger values and become much wider, with a variance of $\sigma^{2}$ ≈ 120 eV${}^{2}$ as compared with the indentations with thermostatted particles ($\sigma^{2}<$ 1 eV${}^{2}$); see Figure 5e.

## 4. Conclusions

In this work, we study the effect that different deterministic and stochastic thermostats have on the mechanical and thermal properties of an indented Ta crystal. We perform MD simulations with unthermostatted and NH- and Langevin-thermostatted particles. The NH thermostats are characterized by the parameter $\omega_{p}$. With $\omega_{p}$ = 100 d*t*, the NH bath effectively acts on the system while the NH thermostat with $\omega_{p}$ = 100,000 d*t* allows only a limited exchange of energy between the system and the bath.

We present a systematic analysis of the work and kinetic fluctuations obtained in two distinct indentation protocols that produce reversible elastic and non-reversible plastic deformations in the crystal. Our main observations are summarized as follows:

1. In our indentation simulations, the system–bath coupling prescribed by the thermostats has a clear effect on the resulting work fluctuations. This is crucial when it comes to obtaining appropriate work statistics that enable free-energy difference calculations by means of the JE and related expressions.
2. In the MD indentations with unthermostatted and NH-thermostatted (with $\omega_{p}$ = 100,000 d*t*) particles, the instantaneous kinetic energy of the system exhibits wild fluctuations when non-reversible plastic deformations are induced in the crystal.
3. The absence or presence of a stochastic thermostat in the dynamics of the particle system respectively represent the cases considered by the Hamiltonian and Langevin derivations of the JE. We find that the differences between the two approaches are substantial and bring about non-negligible effects in the calculation of the left-hand side of the JE. Such differences are clearly observable in the work distributions obtained under the fully reversible elastic protocol.

## Data Availability

The data that support the findings of this study are available from the corresponding author upon reasonable request.

## Supplementary Materials

The following are available online at https://www.mdpi.com/article/10.3390/e24091309/s1, Figure S1: Effect of the NH thermostat frequency $\omega_{p}$ on the indentation load and kinetic energy fluctuations during the plastic protocol with $h_{\max}$ = 9.5 Å, Figure S2: The effect of the damping factor $\gamma_{L}$ on the load-penetration curves and on the time evolution of the kinetic energy of the system, Figure S3: Atomistic snapshots captured during the elastic protocol ($h_{\max}$ = 3.5 Å) with NH-thermostatted particles ($\omega_{p}$ = 100 d*t*), Figure S4: Atomistic snapshots captured during the elastic protocol ($h_{\max}$ = 9.5 Å) with NH-thermostatted particles ($\omega_{p}$ = 100 d*t*) [23,24,25,26,27,28,29,30,31].
