# Supplementary material for: Work and Thermal Fluctuations in Crystal Indentation under Deterministic and Stochastic Thermostats: The Role of System–Bath Coupling
Source: Entropy (Basel). 2022 Sep 15;24(9):1309. doi: 10.3390/e24091309 (PMC9497718; doi:10.3390/e24091309)
Supplement: Supplementary file 1 [file entropy-24-01309-s001.zip › entropy-1824911-supplementary.pdf]

Supplementary information to the manuscript “*Work and Thermal Fluctuations in Crystal Indentation under Deterministic and Stochastic Thermostats: The Role of System-Bath Coupling*”

Javier Varillas[1] and Lamberto Rondoni[2,3]

[1] Institute of Thermomechanics, Czech Academy of Sciences, 18200 Prague, Czechia;
jvarillas@it.cas.cz

[2] Dipartimento di Scienze Matematiche, Politecnico di Torino, 10125 Turin, Italy;
lamberto.rondoni@polito.it

[3] INFN, Sezione di Torino, Via P. Giuria 1, 10125 Turin, Italy

S1. The Nosé-Hoover thermostat

NVT dynamics employ the Nosé-Hoover (NH) chains [1, 2] that introduce k additional degrees of freedom ξ_k in the $6N$-dimensional phase space, thus mimicking the heat transfer from a (large) reservoir, or thermal bath, with temperature T_{ext}.

The time evolution of a phase space point, $\mathbf{\Gamma}\left(\boldsymbol{r}_i, \boldsymbol{p}_i, \xi_1, \dots, \xi_M, p_{\xi_1}, \dots, p_{\xi_M}\right)_{NVT}$, is described by the following EOM [3],

$$\dot{\boldsymbol{r}}_i = \frac{\boldsymbol{p}_i}{m_i} \qquad i = 1, \dots, N, \tag{S1}$$

$$\dot{\boldsymbol{p}}_i = \boldsymbol{f}_i - \frac{p_{\xi_1}}{Q_1} \boldsymbol{p}_i \,, \tag{S2}$$

$$\dot{\xi}_k = \frac{p_{\xi_k}}{Q_k} \quad k = 1, \dots, M \,, \tag{S3}$$

$$\dot{p}_{\xi_1} = \sum_{i=1}^{N} \frac{\boldsymbol{p}_i^2}{m_i} - (N_{\mathrm{f}} + d^2)\mathrm{k_B} T_{\mathrm{ext}} - p_{\xi_1} \frac{p_{\xi_2}}{Q_2} \,, \tag{S4}$$

$$\dot{p}_{\xi_k} = \left(\frac{p_{\xi_{k-1}}}{Q_{k-1}} - \mathrm{k_B} T_{\mathrm{ext}}\right) - p_{\xi_k} \frac{p_{\xi_{k+1}}}{Q_{k+1}} \quad \text{for} \quad k = 2, \dots, M-1 \,, \tag{S5}$$

Here, Q_k is the “mass” of the kth thermostat that tunes the fluctuations of the system’s temperature T [4]. N_{f} is the number of degrees of freedom (=3N), d the system’s dimension, M the number of NH chains, $\mathrm{k_B}$ is the Boltzmann’s constant, and ξ_k and p_k are, respectively, the thermostat variable (extra M degrees of freedom) and conjugate momentum of the kth thermostat. Note that T is not a strictly conserved quantity in the dynamics but, rather, it is a quantity numerically controlled by an external body (i.e., the heat bath modeled by imposing the above degrees of freedom) which fluctuates around the defined value for the bath’s temperature T_{ext} [3].

It is demonstrated in Ref. [4] that the thermostat masses Q_k should satisfy $Q_1 = N_{\mathrm{f}} \mathrm{k_B} T_{\mathrm{ext}} / \omega_{\mathrm{p}}^2$ and $Q_k = \mathrm{k_B} T_{\mathrm{ext}} / \omega_{\mathrm{p}}^2$ (for $k = 2, \dots, M-1$), where ω_{p} is the frequency at which the particles are thermostatted. Then, to evaluate the effect of ω_{p} on the thermostatting properties in our MD indentations, we run additional MD simulations of the plastic protocol with NH-thermostatted particles with distinct values of ω_{p} (see Section 2 of the main text for further details on the computational methodology). Figure S1 shows the resulting indentation load and kinetic energy fluctuations under a wide range of thermostat frequencies ω_{p}, varying from $\omega_{\mathrm{p}} = 1\mathrm{d}t$ to $\omega_{\mathrm{p}} = 100{,}000\mathrm{d}t$, where $\mathrm{d}t = 2$ fs. It is observed that low frequencies ($\omega_{\mathrm{p}} < 10\mathrm{d}t$) lead to wild fluctuations in both temperature and kinetic energy, while large frequencies ($\omega_{\mathrm{p}} > 10{,}000\mathrm{d}t$) result in roughly constant-energy dynamics due to the poor contribution of the thermostat. In light of these results, we adopt $\omega_{\mathrm{p}} = 100\mathrm{d}t$ for the indentations where the particles are strongly coupled with the bath, whereas in the simulations with weak coupling we employ $\omega_{\mathrm{p}} = 100{,}000\mathrm{d}t$. Notice that the above quantity surpasses the time process of the plastic protocol ($\tau_{\mathrm{pl}} = 190$ ps, cf. Fig. 1(c) in the main text).

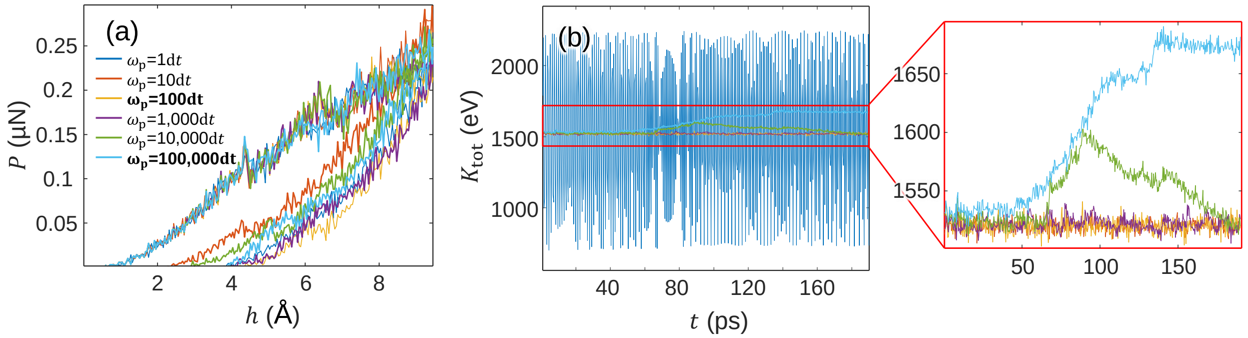

Figure S1. Effect of the NH thermostat frequency ω_p on the (a) indentation load (P) and (b) kinetic energy fluctuations during the plastic protocol with h_{max} = 9.5 Å. The number of NH chains is set to $M = 3$ and the bath's temperature to $T_{ext} = 300$ K. Large frequencies (i.e., $\omega_p > 10{,}000\mathrm{d}t$) lead to a poor contribution of the thermostat in the dynamics. On the other hand, fluctuations in the kinetic energy increase by decreasing ω_p. Extremely small thermostat frequencies ($\omega_p < 10\ \Delta t$) undergo unrealistic NVT dynamics, where wild kinetic fluctuations appear with $\omega_p = 1\ \Delta t$, see (b).

S2. The Langevin thermostat

Langevin dynamics assume that the particles suffer collisions with much lighter ones, which effectively represent the interaction with a heat bath at T_{ext} [5]. This approach mimics the conditions in which the system's particles interact with a background implicit solvent [6]. The collisions are then described by a friction term, $-\boldsymbol{p}_i/\gamma_L$, and a stochastic random force, $\boldsymbol{\eta}(t)$. The Hamiltonian equations of motion (EOM) [7] are coupled to the Langevin equation for the Brownian motion [8], thus leading to the EOM of Langevin dynamics

$$\dot{\boldsymbol{r}}_i = \frac{\boldsymbol{p}_i}{m_i} \qquad i = 1, \dots, N, \tag{S6}$$

and

$$m_i \ddot{\boldsymbol{r}}_i = \boldsymbol{f}_i - \frac{m_i}{\gamma_L} \dot{\boldsymbol{r}}_i + \boldsymbol{\eta}(t)\,. \tag{S7}$$

Here, $\boldsymbol{f}_i$ is the conservative force computed via the usual interparticle interactions. The friction term is a frictional drag or viscous damping term proportional to the velocity of particle i, $\dot{\boldsymbol{r}}_i$. The proportionality constant for each atom is computed as m_i/γ_L, where γ_L is the Langevin damping factor in units of reciprocal time. $\boldsymbol{\eta}(t)$ is a stochastic force due to solvent atoms at temperature T_{ext} that randomly bump into the particle i, and whose magnitude is proportional to $\boldsymbol{\eta}(t) \propto \sqrt{(m_i \mathrm{k_B} T_{ext})/(\gamma_L \mathrm{d}t)}$ [8], as derived from the fluctuation/dissipation theorem [9]. Langevin dynamics allows the system's temperature to be controlled similarly to the NH thermostat, where the probability distribution also approximates the canonical distribution. Notice in Eq. (S7) that the larger the damping coefficient γ_L, the faster the kinetic energy of the system is effectively reduced. In other words, γ_L determines how rapidly the temperature is relaxed.

We then assess the effect of γ_L on the thermostatting properties in our MD indentations by performing additional MD simulations of the plastic protocol with Langevin-thermostatted particles with distinct values

of γ (see Section 2 of the main text for further details on the computational methodology). Figure S2 shows the indentation load and kinetic energy fluctuations from these simulations under varying values of the damping factor γ_{L}, ranging from $\gamma_{\mathrm{L}} = 0.01\ \mathrm{ps}^{-1}$ to $\gamma_{\mathrm{L}} = 10{,}000\ \mathrm{ps}^{-1}$. Given these results, we adopt for our simulations with Langevin-thermostatted particles analyzed in the main text a damping factor of $\gamma_{\mathrm{L}} = 1\ \mathrm{ps}^{-1}$, which produces a coupling (of the particles with the thermal bath) similar to that produced by the NH thermostat under $\omega_{\mathrm{p}} = 100\mathrm{d}t$; compare the K_{tot} fluctuations in Figs. S1(b) and S2(b).

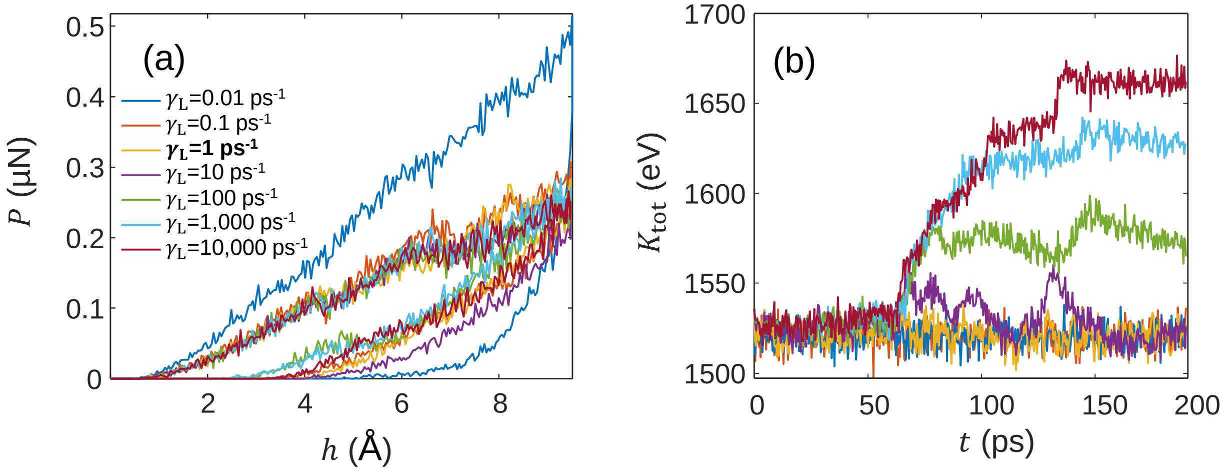

Figure S2. The effect of the damping factor γ_{L} on (a) the load (P)-penetration (h) curves and on (b) the time evolution of the kinetic energy of the system, K_{tot}. The simulations were carried out following the plastic protocol with h_{max} = 9.5 Å.

S3. Reversible vs. non-reversible deformations in our crystal indentations

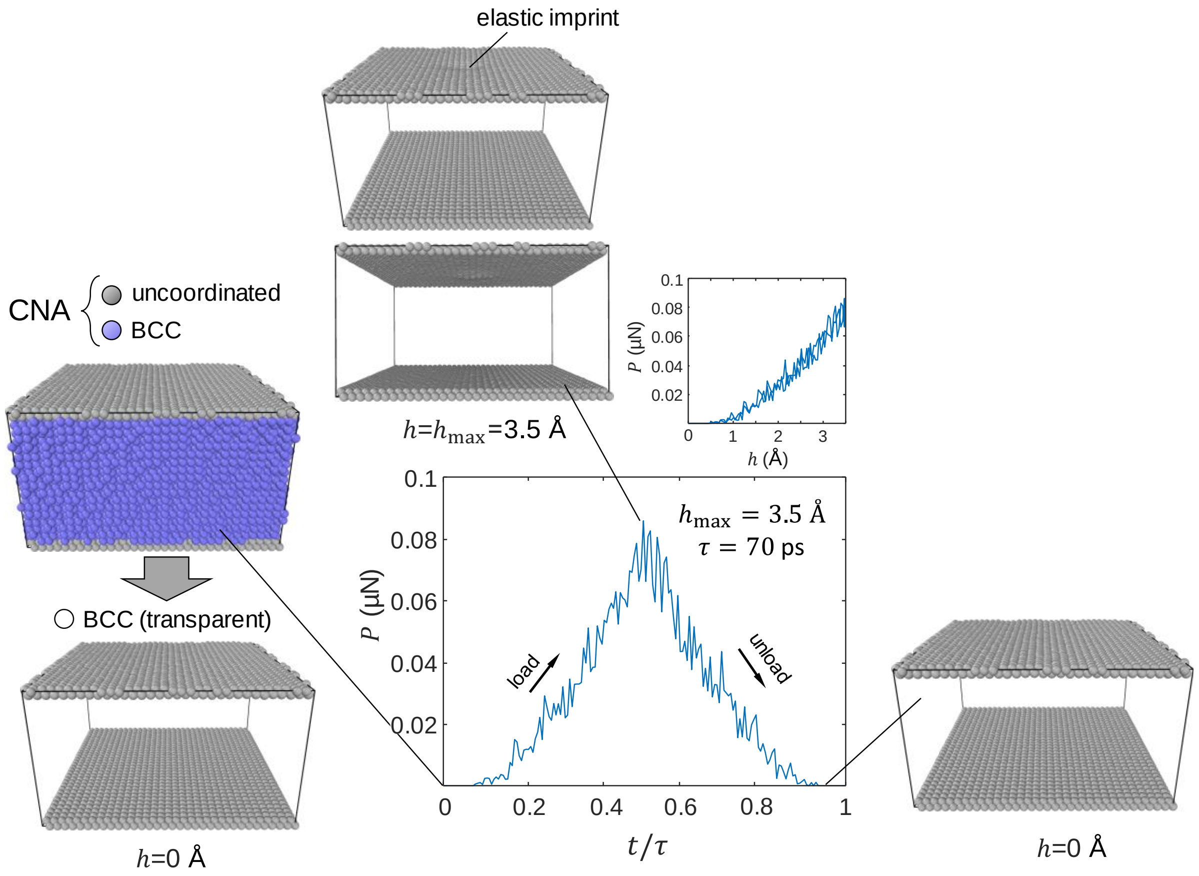

Figure S3. Atomistic snapshots captured during the elastic protocol ($h_{\text{max}} = 3.5$ Å) with NH-thermostatted particles ($\omega_{\text{p}} = 100\text{d}t$). Atomistic visualization was conducted in the OVITO software [10], whereas the common neighbor analysis (CNA) algorithm [11] was employed to calculate the local crystalline structure around the constituent atoms. Note that the uncoordinated (gray) atoms at the top and bottom of the MD cell represent free surfaces. The perturbation during loading leads to the formation of an elastic imprint on the indented surface, which is fully recovered upon unloading.

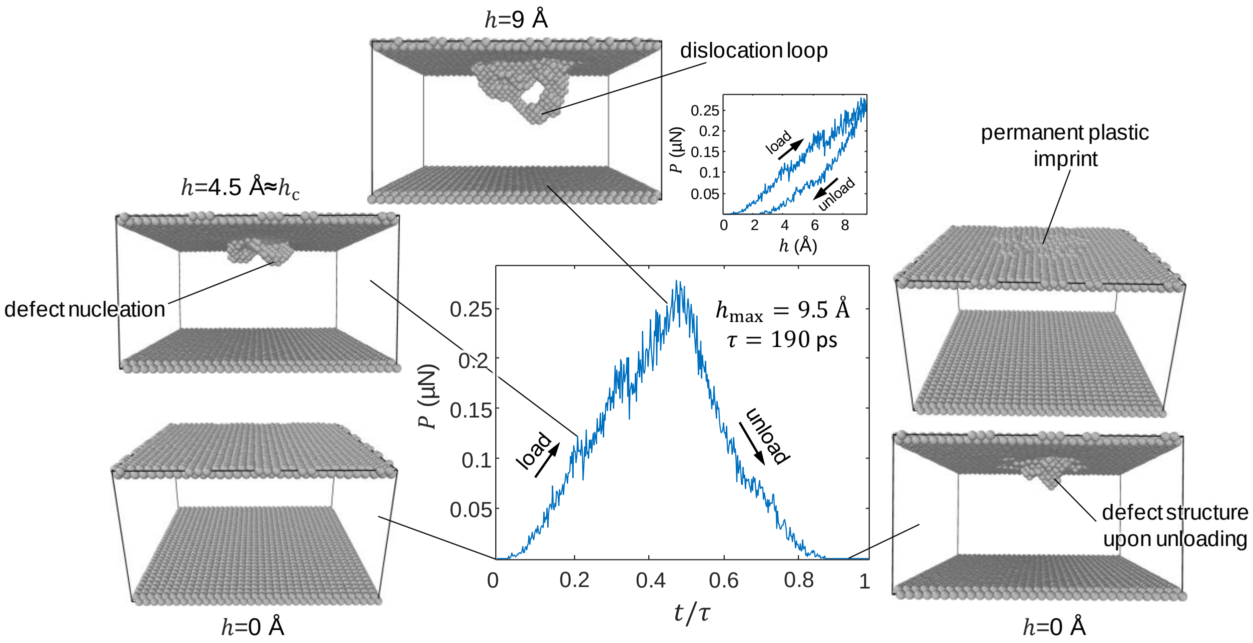

Figure S4. Atomistic snapshots captured during the plastic protocol ($h_{\mathrm{max}} = 9.5$ Å) with NH-thermostatted particles ($\omega_{\mathrm{p}} = 100\mathrm{d}t$). Atomistic visualization was conducted in the OVITO software [10], whereas the common neighbor analysis (CNA) algorithm [11] was employed to calculate the local crystalline structure around the constituent atoms, which allows for the detection of crystalline defects in the Ta BCC crystal associated with uncoordinated (gray) atoms. Note that the uncoordinated atoms at the top and bottom of the MD cell represent free surfaces. In the plastic protocol, the perturbation during loading leads to the generation of crystalline defect at the inception of plasticity at $(P_{\mathrm{c}}, h_{\mathrm{c}})$– see Fig. 4(a) in the main text. With increasing indenter-tip penetrations, the nucleated defects evolve to form a defect structure beneath the indented surface. A plastic imprint is formed during loading that remains in the crystal's surface upon unloading. Similar crystalline processes are observed in the MD indentations with unthermostatted and Langevin-thermostatted particles.

References

[1] W. G. Hoover, "Canonical dynamics: Equilibrium phase-space distributions," Phys. Rev. A, vol. 31, no. 3, pp. 1695-1697, 1985.

[2] S. Nosé, "A unified formulation of the constant temperature molecular dynamics methods," The Journal of Chemical Physics, vol. 81, pp. 511-519, 1984.

[3] W. Shinoda, M. Shiga and M. Mikami, "Rapid estimation of elastic constants by molecular dynamics simulation under constant stress," Phys. Rev. B, vol. 69, no. 13, p. 134103, 2004.

[4] G. J. Martyna, M. E. Tuckerman, D. J. Tobias and M. L. Klein, "Explicit reversible integrators for extended systems dynamics," Molecular Physics, vol. 87, pp. 1117-1157, 1996.

[5] M. P. Allen and D. J. Tildesley, Computer Simulation of Liquids, 2nd ed., O. U. Press, Ed., 2017.

[6] S. Plimpton, A. Thomson, P. Crozier, and A. Kohlmeyer, "LAMMPS massive-parallel atomistic simulator manual, https://lammps.sandia.gov/doc/Manual.html," lammps.sandia.gov, 2022.

[7] W. Cai, J. Li and S. Yip, "1.09 - Molecular Dynamics," in Comprehensive Nuclear Materials , R. J. M. Konings, Ed., Oxford, : Elsevier, 2012, pp. 249-265.

[8] T. Schneider and E. Stoll, "Molecular-dynamics study of a three-dimensional one-component model for distortive phase transitions," Phys. Rev. B, vol. 17, no. 3, pp. 1302-1322, 1978.

[9] D. J. Evans and G. Morris, Statistical Mechanics of Nonequilibrium Liquids, ANU E Press, 1st Ed., 2007.

[10] A. Stukowski, "Visualization and analysis of atomistic simulation data with OVITO the Open Visualization Tool," Modelling and Simulation in Materials Science and Engineering, vol. 18, p. 015012, 2010.

[11] J. D. Honeycutt and H. C. Andersen, "Molecular dynamics study of melting and freezing of small Lennard-Jones clusters," The Journal of Physical Chemistry, vol. 91, pp. 4950-4963, 1987.
